# Editorial: Versatility of algae in addressing global sustainability challenges

**DOI:** 10.3389/fbioe.2025.1728103

**Published:** 2025-11-07

**Authors:** Abdelfatah Abomohra, Klaus von Schwartzenberg, Figueroa L. Felix, Dieter Hanelt

**Affiliations:** 1 Aquatic Ecophysiology and Phycology, Institute of Plant Science and Microbiology, University of Hamburg, Hamburg, Germany; 2 Andalusian Institute for Biotechnology and Blue Development (IBYDA), Experimental Center Grice Hutchinson, Malaga University, Lomas de San Julián, Malaga, Spain

**Keywords:** algal biotechnology, carbon sequestration, microalgae, macroalgae, sustainability, phycochemical

Algae have emerged as one of the most versatile and sustainable bioresources capable of addressing the pressing challenges of the 21^st^ century, including climate change, environmental degradation, resource depletion, and food-energy insecurity. Their exceptional biological diversity, rapid growth rates, and ability to thrive in diverse environments make them uniquely suited for sustainable biotechnological applications. Beyond their ecological roles, algae offer immense potential in carbon sequestration, renewable energy production, nutrient recycling, and the generation of high-value bioactive compounds. Recent advances continue to highlight their pivotal role in fostering a sustainable and circular bioeconomy (Tang et al., 2023; Zaky and Abomohra, 2023; Ende et al., 2024). In this context, this Research Topic brings together five contributions that collectively advance the understanding of algal biotechnology, from fundamental biological insights to applied innovations.

The broad potential of algae in addressing global sustainability challenges has been extensively discussed in the mini-review *“* Dev Das and Bhattarai”. This paper highlights the capacity of algae to mitigate climate change, conserve natural resources, remediate environmental pollutants, and support food and energy security. Microalgae such as *Chlorella* sp.*, Nannochloropsis* sp.*, Botryococcus* sp.*,* and *Spirulina* sp. demonstrate remarkable efficiencies in biomass production, carbon capture, nutrient recycling, and bioenergy generation. Moreover, macroalgae like *Asparagopsis* sp. have proven effective in reducing methane emissions in ruminant livestock, illustrating the role of algae in climate-smart agriculture (Steinhausen et al., 2024). On the other hand, the invasive brown alga *Rugulopteryx okamurae* has rapidly proliferated along the southern European coastline, causing significant ecological and socioeconomic impacts by displacing native species and altering habitat structures (Figueroa et al., 2025). While regulatory frameworks have been introduced to address its invasive spread, studies confirmed that *R. okamurae* contains valuable biomolecules such as terpenoids, alginates, and carotenoids with promising applications in biotechnology, aquafeed, biostimulants, and eco-friendly biomaterials such as bioplastics and packaging materials. These findings underscore how targeted valorization, even in invasive biomass, could contribute to blue and circular economy strategies. Despite these advances, challenges related to scalability, economic feasibility, contamination risks, and regulatory barriers, particularly in food and feed applications, persist, emphasizing the need for targeted research and technological innovation.

Building upon these foundational insights, the review *“* Ashour et al. *”* delves deeper into the potential of microalgae as tools for utilization of atmospheric CO_2_ and bioenergy production. Taking *Chlorella* species as example, their highly efficient carbon-concentrating mechanism (CCM) enables effective CO_2_ sequestration while providing a sustainable feedstock for biohydrogen, biodiesel, bioethanol, biogas, and other bioenergy products. Factors such as pH, temperature, light intensity, nutrient availability, and dissolved oxygen critically influence microalgal CO_2_ uptake and bioenergy conversion efficiency. Additionally, using industrial waste and side streams, such as abattoir wastewater or crude glycerol (Xu et al., 2019; Elsayed et al., 2024), for algae cultivation enhances nutrient recycling, mitigates environmental pollution, and supports low-carbon circular bioeconomy strategies. These findings highlight the promise of algae-based systems as environmentally friendly alternatives to conventional CO_2_ mitigation and energy production methods.

Translating this potential into practical applications requires innovative and sustainable cultivation strategies. The research paper titled “Ende et al.” explored the use of brine and struvite as alternative nutrient sources for cultivating *Arthrospira platensis*. These sustainable media not only supported high biomass productivity but also enhanced c-phycocyanin (C-PC) yields, a valuable pigment with applications in food, cosmetics, and pharmaceuticals. By replacing conventional salts and phosphates with waste-derived nutrients, the study demonstrated that algae cultivation could be more environmentally-friendly and cost-effective, offering scalable solutions for commercial production while promoting circular resource use. This work exemplifies the integration of low-cost cultivation methods with sustainable bioprocessing for industrial and environmental benefits. The implications extend beyond biomass production, as a recent study confirmed that microalgae also exhibit a remarkable capacity for spontaneous desalination with enhanced value-added products (Ebaid et al., 2025), highlighting their potential role in integrated systems for wastewater treatment, brine desalination, and sustainable aquaculture.

Maximizing the production of high-value metabolites in algae requires a combination of environmental manipulation and metabolic engineering (Abomohra and Ende, 2024). In the study “Hu et al.” CO_2_ chemical absorption and microalgae conversion (CAMC) system using *Dunaliella salina* was investigated. Nitrogen stress was applied in combination with exogenous phytohormones to redirect carbon flux toward polysaccharide and β-carotene synthesis. Supplementation with gibberellin under nitrogen-limited conditions enhanced polysaccharide and β-carotene accumulation. This approach not only improved biomass yield but also provided insights into carbon metabolism regulation under stress conditions, suggesting new strategies for integrating CO_2_ capture with the production of economically valuable metabolites.

Complementing this work, the research study on *Chlamydomonas reinhardtii* titled “Mei et al.” investigated how high light and genetic modification influence carotenoid metabolism. In this context, many algal bioactive carotenoids are highly valued for their antioxidant properties and industrial applications (Rahaman et al., 2025). The study reported that high light exposure could positively induce specific carotenoids while negatively affecting others. Furthermore, overexpression of β-carotene ketolase, combined with codon optimization, intron insertion, and chloroplast-targeting peptides, resulted in significant increase in canthaxanthin content compared to wild-type strains. These results illustrate how advanced metabolic engineering, in conjunction with controlled environmental stress, can optimize the production of bioactive compounds in algae, enhancing both yield and economic value.

Taken together, the studies compiled in this Research Topic highlight a continuum of research from fundamental insights into algae metabolism to applied strategies for sustainable cultivation and bioactive compounds production. Algae versatility allows them to simultaneously address environmental, economic, and societal challenges. By combining innovative cultivation methods, metabolic engineering, and integration with circular bioeconomy strategies, algae can become central to sustainable development frameworks that aim to mitigate climate change, conserve resources, and ensure food and energy security. Looking ahead, future research should focus on scaling up these innovative systems while reducing production costs and energy requirements. Multi-disciplinary approaches integrating biotechnology, metabolic engineering, environmental sciences, and industrial process optimization will be key to unlocking the algal full potential. Additionally, harmonized regulatory frameworks and policy support will be essential to facilitate the adoption of algae-based solutions in agri/aquaculture, industry, and environmental management. Public engagement, education, and effective technology transfer will play a critical role in ensuring that these innovations reach communities and industries, accelerating their societal impact. By bridging fundamental research with practical implementation, algae have the capacity to transform global sustainability efforts, providing cleaner energy, healthier ecosystems, and resilient food systems.

In conclusion, algae are not merely bioresources, they are versatile and multifunctional tools for addressing pressing sustainability challenges. From CO_2_ capture and bioenergy production to climate-smart agriculture and circular bioeconomy integration, algae offer unparalleled potential for a sustainable future. The collective insights from the current Research Topic demonstrate both the achievements and opportunities in harnessing algae to meet global environmental, economic, and societal goals. While algae alone cannot solve all sustainability issues, they represent a crucial piece of the larger puzzle, complementing other renewable technologies, policy measures, and societal efforts. Engaging the public, raising awareness, and ensuring effective technology transfer will be essential to translate research advances into practical impact. Continued innovation, coupled with strategic implementation, will ensure that algae contribute meaningfully to shaping a sustainable and resilient future for next generations.
